# Data on a new sensitivity-improved miniaturized label-free electrochemical biosensor

**DOI:** 10.1016/j.dib.2018.01.096

**Published:** 2018-02-03

**Authors:** Yi-Ching Kuo, Chih-Kung Lee, Chih-Ting Lin

**Affiliations:** a Engineering Science & Ocean Engineering, National Taiwan University, Taipei, Taiwan; b Institute of Applied Mechanics, National Taiwan University, Taipei, Taiwan; c Graduate Institute of Electronics Engineering, National Taiwan University, Taipei, Taiwan

## Abstract

This article presents a new sensitivity-improved electrochemical measurement architecture for cardiovascular disease (CVD) diagnosis by detecting CVD biomarkers, S100 beta protein and C-reactive protein (CRP). The new architecture includes a design for a new electrochemical measurement set-up, which improves the reaction conditions of chemical and biological molecules and incorporates a newly biochip design. With the new architecture, electrochemical measurement experiments were undertaken. The results obtained are related to the research article entitled “Improving sensitivity of a miniaturized label-free electrochemical biosensor using zigzag electrodes” [1].

**Specifications Table**

**Table:** 

Subject area	Biosensors
More specific subject area	Electrochemistry
Type of data	Graphs, figures, tables, text files
How data was acquired	Electrochemical analyzer (CHI614D, CH Instrument), Biacore T200 (GE Healthcare)
Data format	Raw
Experimental factors	The proteins at different concentrations were captured by the antibodies based on a 4-ATP or cysteamine modified sensing surface.
Experimental features	1. Development of the process for antibody-antigen interactions. 2. Detection of two kinds of CVD biomarkers, S100 beta proteins and C-reactive proteins, taken as electrochemical impedance measurements.
Data source location	National Taiwan University in Taipei, Taiwan
Data accessibility	Data is with this article
Related research article	Y.-C. Kuo, et al., "Improving sensitivity of a miniaturized label-free electrochemical biosensor using zigzag electrodes," Biosensors and Bioelectronics, 2018.

**Value of the data**

● The data article provides the design concept of an electrochemical measurement set-up.
● The flow rate and the reaction time of the chemical/biological molecules can offer improvement for a more effective sensing surface.
● The electrochemical measurement sensitivity can be improved by adopting an interdigitate-zigzag biochip.
● Two kinds of CVD biomarkers, S100 beta proteins and CRP, can be detected not only on an ATP-based surface but also on a cysteamine-based surface.

## 1 Data

**Fig. 1:**
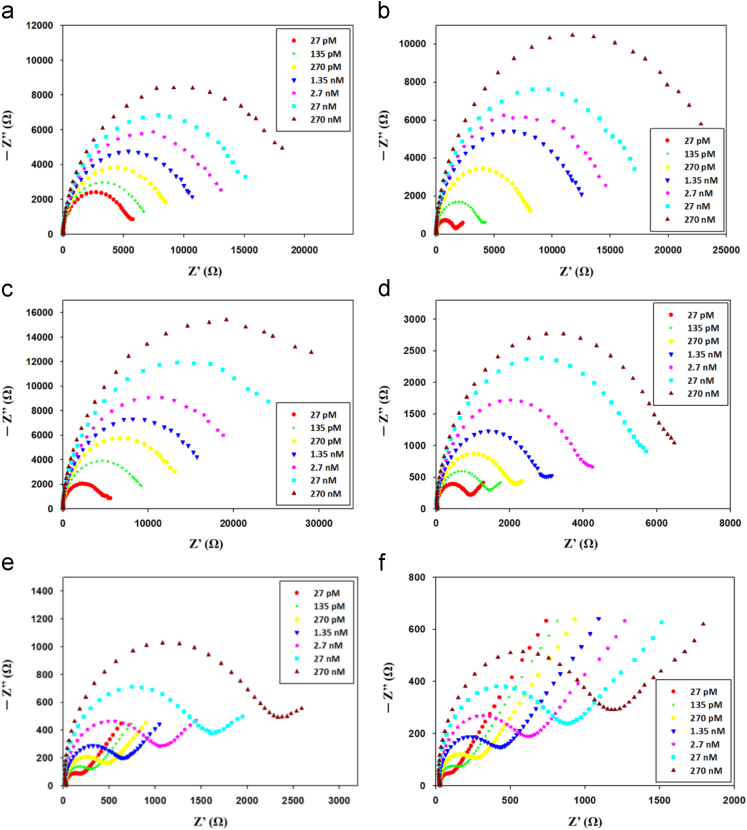
Impedance data of the linker/glutaraldehyde/antibody/ETA&BSA modified electrode in a 1× PBS solution with 1 mM Fe(CN)_6_^3−/4−^ after addition of the target protein at different concentrations: (a) 4-ATP, antiS100, and S100 beta protein in Nyquist form of interdigitate biochip, (b) 4-ATP, antiS100, and S100 beta protein in Nyquist form of interdigitate-semicircle biochip, (c) 4-ATP, antiS100, and S100 beta protein in Nyquist form of interdigitate-zigzag biochip, (d) 4-ATP, antiCRP, and CRP in Nyquist form of interdigitate-zigzag biochip, (e) cysteamine, antiS100, and S100 beta protein in Nyquist form of interdigitate-zigzag biochip, and (f) cysteamine, antiCRP, and CRP in Nyquist form of interdigitate-zigzag biochip.

This article is related to the research article entitled “Improving sensitivity of a miniaturized label-free electrochemical biosensor using zigzag electrodes” [1]. This article presents an improved electrochemical impedance measurement system for cardiovascular disease (CVD) diagnosis by detecting CVD biomarkers, S100 beta proteins and C-reactive proteins (CRP) [2]. Fig. 1 shows the electrochemical impedance data in the Nyquist form for detecting S100 beta protein and CRP. In the new measurement system, three kinds of biochips (e.g. interdigitate biochip, interdigitate-semicircle biochip, and interdigitate-zigzag biochip) were used based on 4-ATP and cysteamine modified architectures.

## 2 Experimental design, materials, and methods

### 2.1 Microfluidic system for electrochemical measurement [3]

**Fig. 2:**
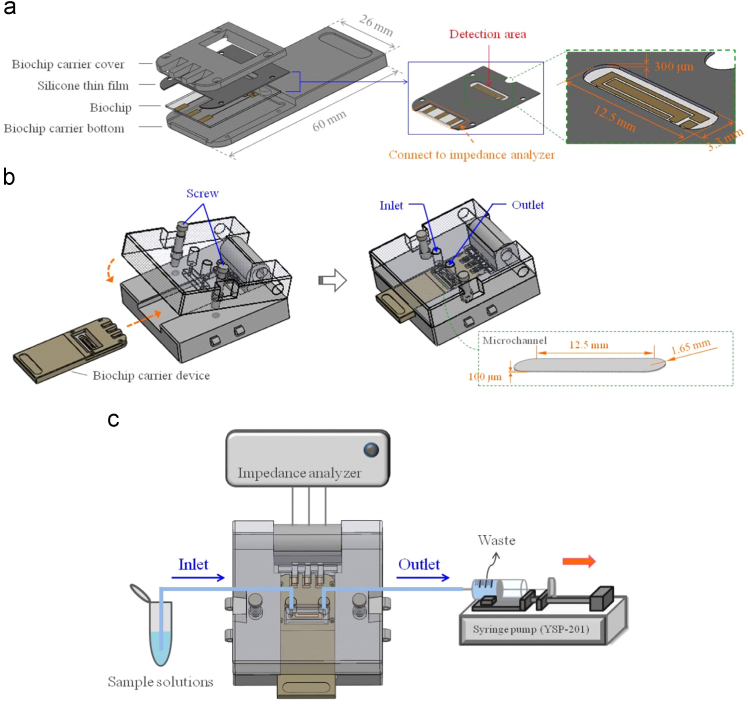
Electrochemical measurement set-up used in this article.

Silicone thin film of 300 μm thickness was used to fabricate the microchannel due to its characteristic properties such as elasticity, malleability, sealability, hydrophobicity, and inactiveness in chemicals. First, the silicone thin film was covered on the biochip. The hollowed area of the silicone thin film formed a closed volume around the electrodes of the biochip as a detection area (see Fig. 2(a)). The cover of the measurement device with an inlet and an outlet was then pressed onto the silicone thin film around detection area by screwing the cover and bottom of measurement device together to let the solutions flow through the microchannel without oozing into the solutions (Fig. 2(b)). Due to the pressing process, the thickness was changed from 300 μm to 100 μm, and a microchannel with a 100 μm height was formed. The closed volume of the microchannel was about 4.98 mm^3^. A syringe pump was used to drive the solutions of the electrochemical measurement system. The impedance analyzer was then linked to the electrodes of the biochip to perform the electrochemical measurements (see Fig. 2(c)).

### 2.2 Modification condition improvement on biochip electrode

**Fig. 3:**
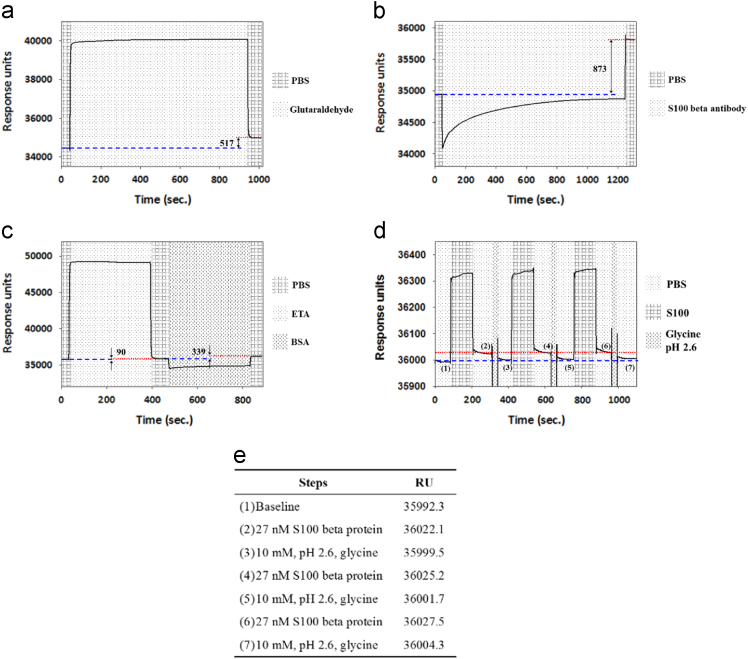
SPR data for antiS100-S100 interaction: (a) during activation, (b) during immobilization, (c) during blocking, (d) during regeneration (Note: red dashed line represents the signal after antigen binding and the blue dotted line represents the signal after regeneration), and (e) RU during regeneration in item (d).

SPR (Biacore T200) was used to determine the reaction conditions and parameters in the electrochemical experiments. A sensor chip (BR-1004-05, GE Healthcare Life Sciences), was used for the SPR measurements. Before modification, a gold sensor surface was ultrasonically cleaned in D.I. water for 3 min. After cleaning, the gold sensor surface was immersed in a 4-ATP solution for 2 h at room temperature, which was used as linker for the SPR measurements. This reaction time of 2 h was enough for the linker to form a pack SAM. The reaction conditions were consistent with that in electrochemical measurements. The gold sensor surface was then rinsed with 75% ethanol and D.I. water and dried using N_2_ stream. After modification of the 4-ATP, the sensor chip was then inserted into the Biacore T200 for the SPR measurements. The S100 beta protein was used as the model protein to find the reaction conditions for experimental processes, and the S100 beta antibody was used to capture the S100 beta protein (Fig. 3).

The glutaraldehyde was first introduced to react with the 4-ATP modified sensor chip. This step was in order to allow the aldehyde functional group to be exposed to the sensor chip surface for the following antibody immobilization. The flow rate was set at 5 μl/min. After reacting for 15 min, when the SPR signal almost reached saturation, the change of the SPR signal was about 517 response unit (RU) (see Fig. 3(a)). The reaction time for the glutaraldehyde injection was thus set at 15 min. The 111 nM S100 beta antibody was then introduced to bind to the aldehyde exposed to the sensor chip surface. The flow rate was also fixed at 5 μl/min. After reacting for 20 min, when the SPR signal almost reached saturation, the recorded measurement was about 873 RU, as depicted in Fig. 3(b). The reaction time for adding the S100 beta antibody was thus set to be 20 min. To avoid unwanted binding between the aldehyde, which was not replaced by the S100 beta antibody, and the NH_2_ functional group of S100 beta protein, ethanolamine (ETA) and bovine serum albumin (BSA) were introduced to block the sensor chip. The flow rate was also fixed at 5 μl/min. For both ETA and BSA, the reaction times were both 6 min, which was enough to allow the SPR signal to reach saturation (Fig. 3(c)). The changes recorded were 90 RU and 339 RU for ETA and BSA, respectively.

**Fig. 4:**
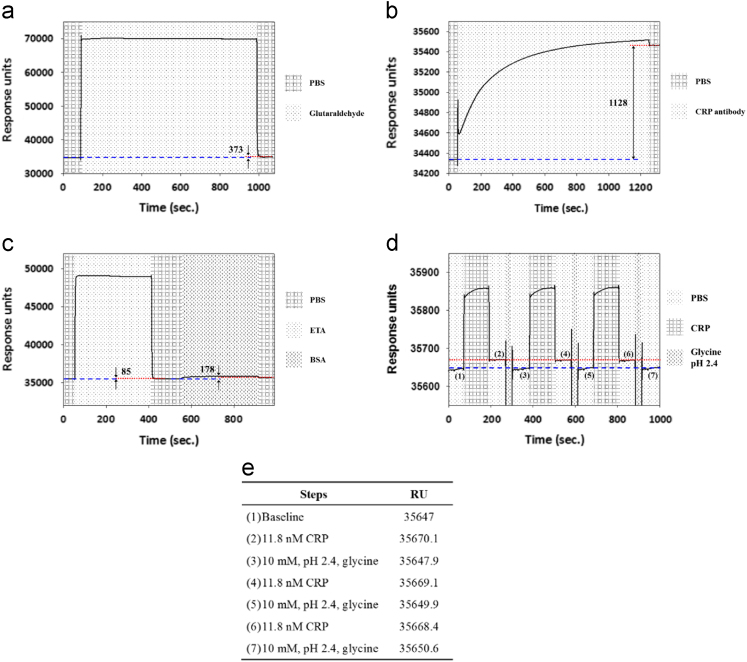
SPR measurement data for the antiCRP-CRP interaction: (a) during activation, (b) during immobilization, (c) during blocking, (d) during regeneration (Note: red dashed line represents the signal after antigen binding and the blue dotted line represents the signal after regeneration), and (e) RU during regeneration in item (d).

From the reaction conditions of the antiS100-S100 interaction process, the obtained SPR signals of the experimental processes for the antiCRP-CRP interactions are shown in Fig. 4. The reaction conditions for all the processed steps were almost enough for the SPR signal to reach saturation.

The regeneration conditions for interrupting the bond between the S100 beta antibody and S100 beta protein were also found by using SPR measurements. A 4-ATP/glutaraldehyde/antiS100/ETA&BSA modified chip was used. The 27 nM S100 beta protein followed by the regeneration buffer was then introduced to observe the changes of the SPR signal (Fig. 3(d)). When the S100 beta protein was injected to interact with S100 beta antibody at 25 μl/min for 2 min, the SPR signal increased. The 10 mM glycine at pH 2.6 was then introduced at 25 μl/min for 30 s, and the SPR signal decreased. This procedure (S100 beta protein followed by the regeneration buffer) was repeated three times. The detailed information of RU during regeneration steps for the S100 beta protein is shown in Fig. 3(e).

To quantify the regeneration data, a recovery ratio was defined:

Recoveryratio=RUProtein-RURegenerationRUProtein-RUOriginal

where *RU_Protein_* is the *RU* after adding the protein, *RU_Regeneration_* is the *RU* after adding the regeneration buffer, and *RU_Original_* is the *RU* before adding the protein. During the first period of Fig. 3(d), the recovery ratio was about 76%. For the second and third period, the recovery ratios were about 91% and 90%, respectively. Moreover, during the three periods, the S100 beta protein produced 29.8, 25.7, and 25.8 *RU* at the first, second, and third run. There was no obvious change in these three runs. It illustrated that, during these three experimental periods, the regeneration condition was applicable to interrupt the binding between S100 beta protein and S100 beta antibody and did not destroy the sensing functional group. This regeneration condition was then used in electrochemical experiments for interrupting the binding between the S100 beta antibody and S100 beta protein during detection of the different concentrations of the S100 beta protein.

The regeneration conditions to interrupt the bond between antiCRP and CRP were also found by using SPR measurements. The 4-ATP/glutaraldehyde/antiCRP/ETA&BSA modified chip was used, and the 11.8 nM CRP followed by the regeneration buffer was then introduced to observe the changes of the SPR signal. When the CPR was injected to interact with the antiCRP at 25 μl/min for 2 min, the SPR signal increased, followed by a 10 mM glycine at pH 2.4 introduced at 25 μl/min for 30 s, resulting in a decreased SPR signal. This procedure (CRP followed by the generation buffer) was repeated three times. The detailed information of the RU during regeneration steps for the CRP are shown in Fig. 4(e).

During the first period of Fig. 4(d), the recovery ratio was about 96%. For the second and third period, the recovery ratios were 91% and 96%, respectively. Moreover, during the three periods, the CRP produced 23.1, 21.2, and 18.5 RU at the first, second, and third run. There was no obvious change in these three runs. It illustrates that, during these experimental periods, the regeneration condition was suitable to interrupt the binding between the CRP antibody and CRP, and did not destroy the sensing functional group. This regeneration condition was then used in the electrochemical experiments for interrupting the binding between the CRP antibody and CRP for detection of the different concentrations of CRP.
